# Direct observation of liquid nucleus growth in homogeneous melting of colloidal crystals

**DOI:** 10.1038/ncomms7942

**Published:** 2015-04-21

**Authors:** Ziren Wang, Feng Wang, Yi Peng, Yilong Han

**Affiliations:** 1 Department of Physics, Hong Kong University of Science and Technology, Clear Water Bay, Hong Kong SAR, China

## Abstract

The growth behaviour of liquid nucleus is crucial for crystal melting, but its kinetics is difficult to predict and remains challenging in experiment. Here we directly observed the growth of individual liquid nuclei in homogeneous melting of three-dimensional superheated colloidal crystals with single-particle dynamics by video microscopy. The growth rate of nucleus at weak superheating is well fitted by generalizing the Wilson–Frenkel law of crystallization to melting and including the surface tension effects and non-spherical-shape effects. As the degree of superheating increases, the growth rate is enhanced by nucleus shape fluctuation, nuclei coalescence and multimer attachment. The results provide new guidance for the refinement of nucleation theory, especially for the poorly understood strong-superheating regime. The universal Lindemann parameter observed at the superheat limit and solid–liquid interfaces indicates a connection between homogeneous and heterogeneous melting.

## Abstract (web summary)

Monitoring crystal melting at a single-atom level is challenging because of the small spatial and temporal scales involved, especially for a bulk process. Wang *et al*. report the melting dynamics of a colloidal crystal with emphasis on the growth of critical nuclei upon different degrees of superheating.

## Graphical abstract

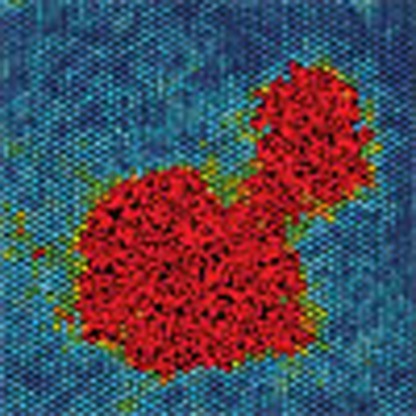

Crystal melting and freezing in three dimensions (3D) are important first-order phase transitions that proceed through the nucleation mechanism, but a first-principle theory remains unavailable^1^. In the phenomenological classical nucleation theory (CNT), the nucleus of the product phase forms spontaneously from the parent phase under thermal fluctuations with the free energy^2^


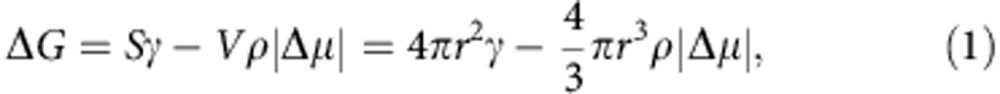


where *r* is the effective radius of the nucleus with surface area and volume *V* is the surface tension, *ρ* is the number density of particles in the nucleus, and |Δ*μ*| is the chemical potential difference between the parent and product phases. The free energy reaches a maximum 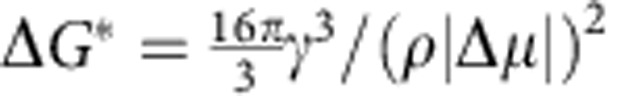 at the critical size r*=2*γ*/(*ρ*|Δ*μ*|). If the parent phase is a solid, equation 1 contains an additional strain energy that is proportional to *V* and can be absorbed into the volume term. When a subcritical nucleus fluctuates and crosses the energy barrier, it becomes a post-critical nucleus and tends to grow irreversibly. However, equation 1 cannot predict the kinetics of the nucleation process. In CNT, the predictions of kinetics are based on several approximations, which usually hold only at weak supersaturation, and the quantitative nucleation behaviours of melting and freezing at strong supersaturation are largely unknown.

As few experiments on homogeneous nucleation in melting have been reported so far, we will compare our results with the homogeneous nucleation in crystallization. Crystal melting and freezing share some common features, but they also differ in many aspects^3^. For example, the nucleation rate increases monotonically with the degree of supersaturation in melting, whereas it peaks before dropping to zero at the glass transition point in freezing. Liquids can be easily supercooled for crystallization, but it is difficult to superheat crystals because they start melting from the surfaces or gain boundaries once they reach the melting point^4^,^5^. Lasers have been used to superheat the interior of atomic crystals^6^, but it results in catastrophic melting and cannot show the kinetics of quasi-static phase transitions. Some crystals covered by higher melting point materials^7^,^8^,^9^ or by antimelting proteins^10^ can be superheated, but they often melt from the surfaces before reaching a deep superheating. Light scattering techniques can detect small crystallites nucleated in liquids, but lack the sensitivity to discern small liquid nuclei in large crystals. As a result, nucleation in melting has been nowhere near as thoroughly investigated as crystallization.

The nucleation process of melting can be divided into three stages: (I) The incubation stage in which the superheated crystal remains metastable without forming critical liquid nuclei. However, nucleation precursors such as defects^11^,^12^ or particle-swapping loops^13^,^14^ may form and trigger the formation of liquid nuclei. (II) The formation of critical nuclei. (III) The growth stage of post-critical nuclei. Stages (I) and (II) have been simulated^15^,^16^, but not stage (III), which requires large systems. Experimentally, the nucleation process can hardly be observed at the single-particle level in atomic systems due to their small spatial and time scales, especially the nucleation process inside the bulk.

Colloids are outstanding model systems for studying phase transitions because micrometre-sized particles can be directly visualized and their dynamics can be measured inside the bulk using optical video microscopy^17^,^18^. In the intensively studied colloidal crystallization, video microscopy experiments have revealed important microscopic details and insights about stages I and II of nucleation by video microscopy, but stage III has only been studied by light scattering, which only provides ensemble-averaged information. Crystallizations have been studied by breaking colloidal crystals into a supercooled liquid with brutal force and monitoring the latter's evolution back to the equilibrium crystalline phase^19^,^20^,^21^. By contrast, the study of melting requires a *tunable* colloidal system because its initial state needs to be an ordered crystal^14^,^22^,^23^. Moreover, crystals usually melt heterogeneously from the surfaces or grain boundaries once they reach the melting point, and fail to enter the superheated state. We solved these challenges using a combination of diameter-tunable microgel colloidal spheres^5^ and the local optical-heating technique^14^ so that stages I and II of the nucleation can be directly observed at the single-particle level for the first time^14^.

In the present work, we investigated stage III of the post-critical nucleus growth in a similar experimental system, which provides the first direct visualization of the later stage of nucleation in melting at the single-particle level. We studied the effects of nucleus size, shape, coalescence and surface tension on the nucleus growth rate in the whole range of superheating, that is, from the melting point to the superheat limit. We theoretically modified the classical Wilson–Frenkel (WF) law for the nucleus growth in crystallization to the case of melting, which fits the measured nucleus growth rates well at weak superheating. At stronger superheating, more effects beyond the CNT was observed, including the rotation of a nucleus because of the anisotropy of the crystal surface tension, the coalescence of nuclei through neck-formation and multimer attachment.

## Results

### Nucleus growth law

**Figure 1:**
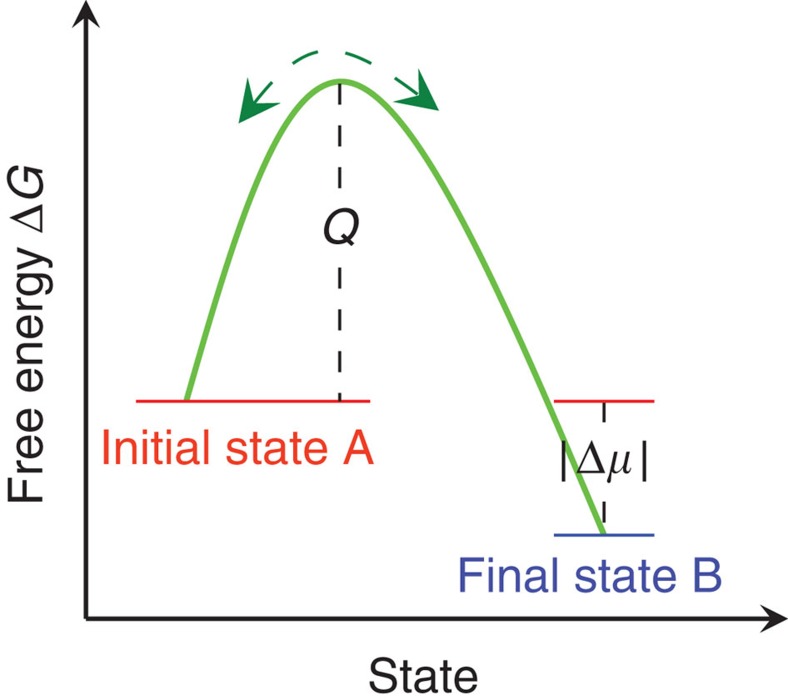
A general barrier-crossing process. *Q* is the activation energy for state A. |Δ*μ*| is the chemical potential difference between the two states.

The nucleus growth rate *v*≡d*r*_eff_/d*t*^24^, where the effective nucleus radius 
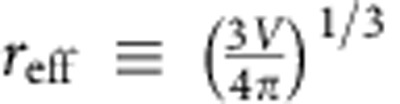
. Here we follow the derivation of the nucleus growth rate in crystallization and derive a similar expression for melting based on the general energy barrier-crossing process^25^,^26^ as shown in Fig. 1. The rate at which a particle transforms from state A to state B is 
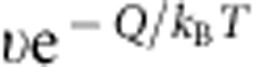
, where *υ* is the collision frequency to jump over the free energy barrier with height *Q* separating states A and B. Similarly, the transformation rate from B to A is 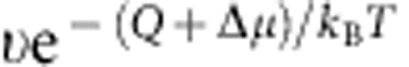, where Δ*μ* is the free energy difference between states B and A (see Fig. 1). In nucleation, A and B represent a parent-phase and a product-phase state, respectively, for a particle on the nucleus surface. Hence, the net growth rate of a nucleus is


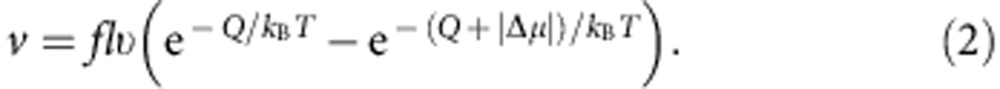


The rate of success *f* is assumed to be constant, that is, independent of the degree of supersaturation^27^. 
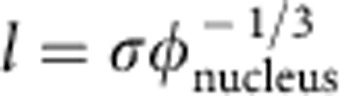
 is the thickness of a particle layer^25^,^26^,^27^. A particle at the nucleus interface has the probability 
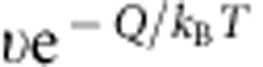
 of jumping out of the cage formed by its nearest neighbours per unit time and the out-of-cage motion has a displacement *l*, hence 
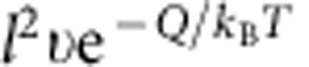
 corresponds to the mean-square displacement (MSD) per unit time and is equivalent to 6*D*′^26^. The diffusion coefficient *D*′ at the liquid–crystal interface is usually not measurable and is approximated as *D* in liquid^26^,^28^.

Based on the above analysis, equation 2 becomes 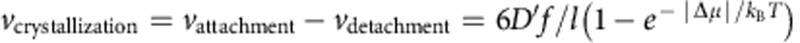, where *v*_attachment_ refers to the rate at which liquid particles attach to crystals. This is the traditional WF growth law or the normal growth law for crystallization^29^,^30^. In melting, state A is crystal, state B is liquid and 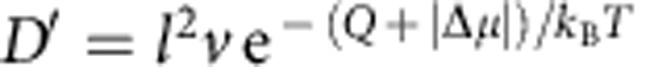 because the diffusion coefficient always corresponds to the activated energy in the liquid phase. Hence, equation 2 for melting becomes 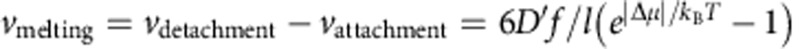. The nucleus growth in crystallization and melting can be combined into a general form:


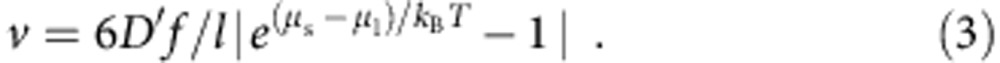


### Experiment

We used thermally sensitive poly(*N*-isopropylacrylamide) (NIPA, NIPAM or pNIPAM) microgel spheres^5^,^31^, whose diameter *σ* changes linearly from 0.80 μm at 23.9 °C to 0.73 μm at 28.1 °C (Supplementary Fig. 4). As the definition of diameter for a soft sphere is ambiguous, we define the diameter such that the melting point is the same as that of hard spheres (*φ*_m_=54.5%)^14^. By this definition, the measured freezing volume fraction *φ*_f_=0.490, which is very close to 
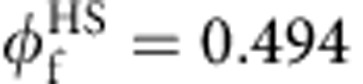
 of hard spheres^32^. NIPA spheres were little charged with short-range steric repulsions in an aqueous butter solution of 0.1 mM acetic acid (see the measured pair potentials *u*(*r*) at different temperatures in refs 23, 33). The NIPA colloidal suspension was flowed into an 18 × 3 × 0.2 mm^3^ glass channel and self-assembled into a 3D face-centred-cubic (fcc) colloidal crystal. Before the experiment, we cycled the temperature near the melting point to anneal some small defects away and release possible pressure gradient. The annealing of the flow and the temperature cycling results a single fcc crystal or a polycrystal with only a few domains. The (111) lattice plane of the crystal was parallel to the glass walls and imaged in the focal plane. The particle density and refractive index closely match to those of water, thus allowing us to directly observe particles inside the 350-layer-thick bulk crystals under bright-field microscopy^5^.

**Figure 2:**
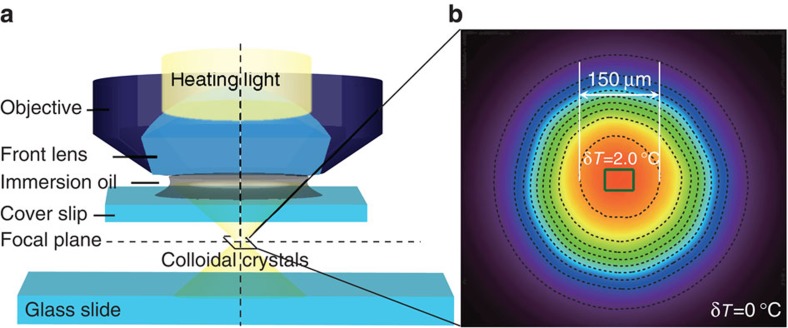
The optical heating. (**a**) The schematic of the local optical heating. (**b**) The measured temperature profile in the focal plane. The contour spacing is 0.2 °C. The temperature in the central region is 2 °C higher than the ambient temperature. The green rectangle at the centre is the field of view.

To avoid heterogeneous melting from the surfaces and defects, we used a beam of light from a mercury lamp to uniformly heat 10^7^ particles inside a defect-free region^14^ (see Fig. 2 and Supplementary Movie 1). We rapidly heated the crystal region above the melting temperature to produce a superheated metastable crystal, and then monitored its evolution into the equilibrium liquid. The heating effect of the light, *δT*=2.0 °C, was stabilized 2 s after the light was turned on (Supplementary Fig. 5). Therefore, the observations were made under a steady state with constant pressure and volume fraction^14^. Note that the phase behaviour of a colloidal system is controlled by volume fraction *φ*, which plays a similar role of inverse temperature in atomic systems. The ambient temperature *T*_amb_ of the whole sample was controlled by the objective heaters (Bioptechs) at 0.1 °C resolution (that is, 0.23% volume fraction). We fixed *δT* and tuned *T*_amb_ to obtain the desired temperature *T*_amb_+*δT* in the region of interest. The crystal was superheated when Δ*T*≡*T*_amb_+*δT*−*T*_m_>0 (that is, the degree of superheating Δ*φ*≡−(*φ*_amb_−*δφ*−*φ*_m_)≡*φ*_m_−*φ*>0), where *T*_m_ is the crystal melting temperature. The heated volume could be tuned by adjusting the iris. The heating light was focused by the objective lens, thus the heating effect was the strongest in the object plane. The heating profile in Fig. 2b was measured from an aqueous solution of yellow fluorescein (0.01% by weight) in a cell 5-μm thick. The brightness of a fluorescent solution is proportional to the light intensity and the heating effect^14^,^34^. The temperature profile was further confirmed from the size of the liquid region after setting the temperature at the centre of the heated area to *T*_m_+0.2 °C. After 2 h of equilibration, the liquid region stabilized to a drum-like shape *π*(75 μm)^2^ in area in the focal plane and −50 μm<*z*<+60 μm in the *z* direction. The temperature in this stable liquid region was quite uniform (<0.2 °C), partly because the scattering from colloids caused the light to smear.

We monitored the middle cross-section of the nucleus during its growth by taking a scan in the *z*-direction every 20–300 s, with each scan lasting less than ∼5 s (Supplementary Movie 2). We thus measured the nucleus volume and shape by integrating the cross-sectional areas and circumferences layer by layer along the *z* direction, respectively. The charge-coupled device camera operated at 15 frames per second. Particle positions were identified using standard image-analysis algorithms^35^.

We label each particle with {*x*_*i*_,*y*_*i*_,*ψ*_6*i*_,*L*_*i*_,*t*}. Here (*x*,*y*) is the position of the particle *j* at time *t*. For a triangular lattice, the sixfold bond-orientational order parameter 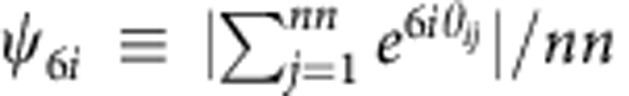 where *θ*_*j*_ is the orientation of the bond between the particle *i* and its neighbour *j*, and *nn* is the number of nearest neighbours^23^. The 3D Lindemann parameters, *L*, in Figs 4, 5, 6, 7 were computed from the measured two-dimensional MSD: 
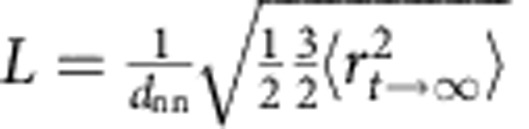
, where *d*_nn_ is the nearest-neighbour distance measured from the first peak position of the radial distribution function and 
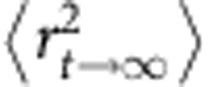
 is the asymptotic value of the two-dimensional MSD^5^. The MSD reaches a plateau due to the caging of neighbouring particles in less than 2 s (refs 5, 14), hence *L* is computed from the ±2 s trajectory around *t*. 
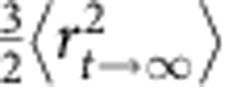
 is the 3D MSD if we assume that particle fluctuations were isotropic. The factor 1/2 arises from the fact that the Lindemann parameter describes the displacement relative to the equilibrium position, whereas the MSD describes the displacement relative to a previous time. Low *ψ*_6*i*_ reflects the local disordered structure, whereas high *L* corresponds to large vibration amplitude. We identified liquid-like particles as those with *ψ*_6*i*_<0.6 and *L*>0.2 so that solid-like particles at the defect with low *ψ*_*6i*_ and low *L* can be screened out^14^.

The nucleation at weak supersaturation is difficult to measure because the induction time taken for a post-critical nucleus to form is extremely long. To study the weak superheating regime, we first burned a post-critical nucleus with a strong heating light, then lowered the heating to the weak superheating regime and monitored the nucleus growth (Supplementary Movie 1). This trick has also been used in simulations of atomic crystal melting^3^,^36^. It enabled the study of stage III, but not the earlier stages I and II at weak superheating. More experimental details are available in the Methods.

We distinguished among four superheating regimes by their nucleus growth behaviours: weak superheating (the degree of superheating Δ*φ*≡*φ*_m_−*φ*≲0.025, where the melting volume fraction *φ*_m_=0.545), intermediate superheating (0.025≲Δ*φ*≲0.05), strong superheating (0.05≲Δ*φ*≲0.06) and very strong superheating (0.06≲Δ*φ*≲0.09). Δ*φ*=0.09 is the measured superheat limit^14^ at which the superheated crystal turns from a metastable state to an unstable one and collapses immediately in zero induction time.

### Weak superheating Δ*φ*≲0.025

**Figure 3:**
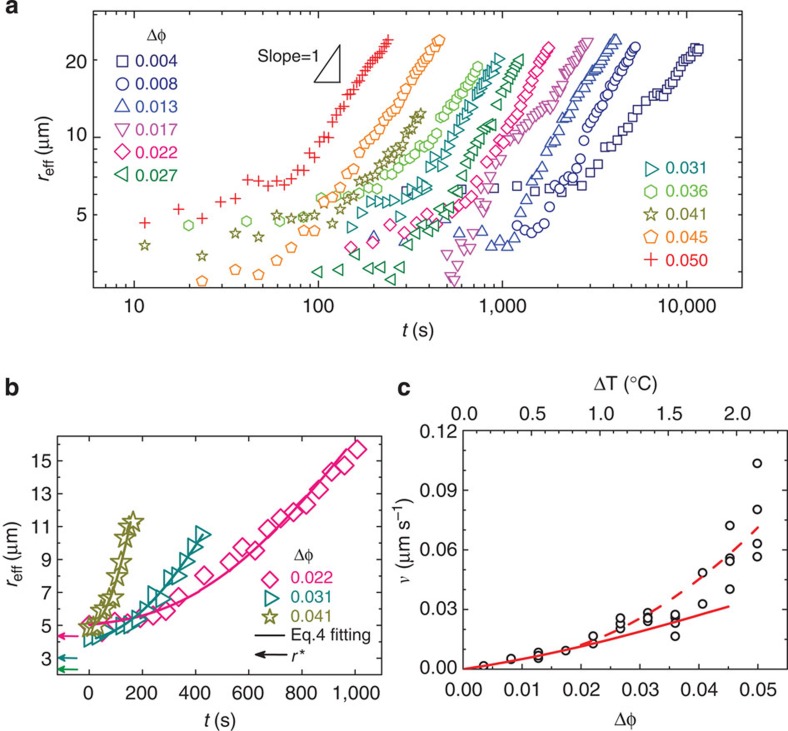
Nucleus growth fitted by the generalized growth laws. (**a**) The typical growth of the effective nucleus radius at different degrees of superheating. Some curves are slightly shifted in time for better display. The shift in time is comparable to the thermal fluctuation of the induction time and much shorter than the measured time range. (**b**) The blowup of **a** with fewer curves for clarity. Solid curves are the fittings of the time integral of equation 4. (**c**) Measured growth rate *v* of large nuclei (circles) fitted by equation 3 multiplied by a prefactor *κ*=1 (solid curve) or *κ*(Δ*φ*)=1+0.2Δ*φ*+400Δ*φ*^2^ (dashed curve). Each circle represents an individual experimental trial.

At weak superheating, we observed that the effective nucleus radius *r*_eff_ increases linearly with time when *r*_eff_>2r* (Fig. 3a). The nucleus growth rate *v* increases with the degree of superheating (Fig. 3c). To fit *v*(Δ*φ*) in Fig. 3c with equation 3, we need to substitute *D*′(Δ*φ*) and *μ*_s_(Δ*φ*)−*μ*_l_(Δ*φ*) into equation 3. As the NIPA particles have almost the same phase behaviour as hard spheres^5^,^14^, we estimate *μ*_s_ and *μ*_l_ from the equations of state for the hard-sphere crystal and fluid, respectively^37^,^38^ (Supplementary Fig. 1 and Supplementary Note 1). In the conventional WF law, *D*′ at the solid–liquid interface is not measurable, and is therefore approximated as the diffusion coefficient in the bulk liquid. It is controversial whether the long-time or the short-time diffusion coefficient should be used in crystallization^39^,^40^. For hard-sphere liquids dispersed in water, the empirical long-time *D*_L_(*φ*)=*D*_0_(1−*φ*_l_/0.58)^1.74^ (refs 41, 42) and short-time *D*_S_(*φ*)=*D*_0_(1−*φ*_l_/0.64)^1.17^ (refs 39, 41), where the Stokes–Einstein relation 
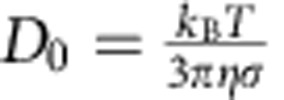
 is the diffusion coefficient of a single particle in the dilute limit and *η* is the viscosity of water. Equation 3 fits the weak superheating regime well in Fig. 3c and the single fitting parameter *f*=0.33 and 0.08 for the long-time and the short-time diffusion coefficients, respectively. Compared with the value of *f*=0.27 obtained from the simulation of crystallization in atomic systems^28^, we found that the long-time *D*_L_ yields a more reasonable *f* in melting.

Note that our equation 3 for melting differs from the traditional WF law for crystallization. The nucleus growth rate increases with the degree of superheating at a higher rate than linearly in melting according to equation 3, but at a lower rate than linearly in crystallization according to the WF law (Supplementary Fig. 3). Their difference arises from the facts that (i) particles can only attach to appropriate lattice sites of a crystalline nucleus in crystallization, but they can attach anywhere in the liquid nucleus in melting; (ii) the diffusion coefficient always pertains to the liquid phase, that is, the parent phase in crystallization and the product phase in melting. The WF law has been thoroughly tested for crystallizations in various atomic^28^ and colloidal systems^43^,^44^,^45^. For melting, however, the nucleus growth rate has not been measured because light scattering cannot properly resolve small liquid nuclei embedded in large crystals.

Equation 3 and the WF law are based on the following four assumptions^24^,^40^: (i) There are always plenty of particles around the nuclei to form the product phase so that the nucleus growth is determined by the incorporation rate of particles into the nucleus surface, that is, an interface-reaction-limited growth characterized by d*V*/d*t*∝*S*. Consequently, *r*∼*t* as confirmed in Fig. 3a. By contrast, vapour condensation or precipitation may deplete product-phase particles near the nuclei so that the nucleus growth is limited by the diffusion of the particles from the bulk to the interface, that is, a diffusion-limited growth described by *r*∝*t*^1/2^. (ii) The nucleus is not faceted and its surface is rough so that the growth takes place without preferential sites. (iii) The nucleus grows through the attachment and detachment of individual particles (monomers) rather than the collective attachment and detachment of multiple particles (multimers)^46^. (iv) The growth applies to a single nucleus without nuclei coalescence. We found that assumptions (i) and (ii) hold well in the whole superheating range, but assumptions (iii) and (iv) break down at deeper superheating.

### Intermediate superheating 0.025≲Δ*φ*≲0.05

At intermediate superheating, the nucleus growth behaviour is qualitatively similar to that at weak superheating, but equation 3 (solid curve in Fig. 3c) underestimates the measured growth rate because (i) *f* may depend on Δ*φ*, (ii) the long-time *D*_L_(Δ*φ*) in bulk liquid may not accurately reflect *D*′(Δ*φ*) at the solid-liquid interface and (iii) multimers rather than monomers may attach to the nucleus. These effects can be accommodated in equation 3 by adding a prefactor *κ*(Δ*φ*)=1+0.2Δ*φ*+400Δ*φ*^2^ from the fitting (the dashed curve in Fig. 3c).

### Effects of surface tension and nucleus shape

The measured *v* follows equation 3 only after the early stage of nucleation. At the early stage of nucleation, however, we found that *v* is strongly affected by the surface tension and the nucleus shape. Small nuclei have large surface-to-volume ratios, hence their surface tension effect is non-negligible. Surface tension should suppress nucleus growth, which is confirmed by the smaller slope at small *r* in Fig. 3a,b. Using r*=2*γ*/(*ρ*|Δ*μ*|) and *φ*=*ρπσ*^3^/6, we account for this effect by adding 
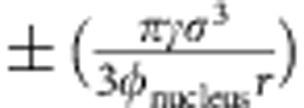
 to the exponent of equation 3 to ensure the nucleus growth rate is zero at the critical size r*, that is, with equal chance of growing and shrinking.

Next, we incorporate the influence of a non-spherical nucleus shape into equation 3. For a non-spherical nucleus, we can introduce a shape factor 
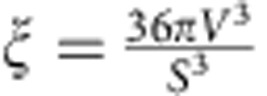
 so that both the nucleus volume 
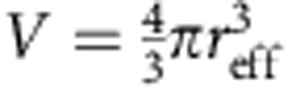
 and the surface area 
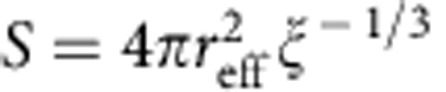
 in equation 1 can be accurately expressed. *ξ*=1 for a sphere and<1 for a non-sphere.

Adding both the surface tension effects and the non-spherical-shape effects to equation 3 gives (Supplementary Note 2)


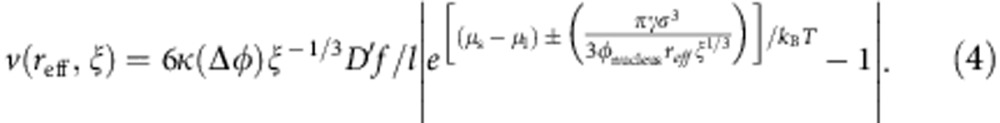


The +sign in equation 4 has been used for crystallization elsewhere^40^ and the −sign should be used for melting since *μ*_s_−*μ*_l_ changes the sign. For nuclei with large *r*, the added term 
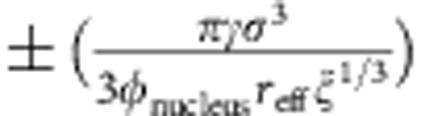
 is negligible because the bulk energy dominates over the surface energy. The time integral of equation 4 fits the curves in Fig. 3a,b nicely in both short-time and long-time regimes. The fitting of the nonlinear short-time regime in Fig. 3b yields *γ*=(0.42±0.03) *k*_B_*T*/*σ*^2^ at 0.02≲Δ*φ*≲0.04, which is less than *γ*=(0.66±0.025) *k*_B_*T*/*σ*^2^ of the equilibrium liquid–crystal interface obtained from the simulation of hard spheres at the melting point^47^. This is reasonable because the volume fraction of a superheated crystal is closer to that of liquid than a non-superheated crystal. The fitted *γ* is averaged over different lattice planes on the surface of a nucleus. *γ* in different planes appear to be similar as we did not observe obvious facets. This is in accordance with the simulation of hard spheres, which found that *γ* of the solid–liquid interfaces has small (<4%) anisotropy in different lattice planes for fcc crystals^47^. Based on the fitted *γ*, we can derive the critical radius r*(*ξ*=1)≈0.21*σφ*_m_/Δ*φ* for a spherical nucleus and the free energy barrier ΔG*=0.023*k*_B_*T*/Δ*φ*^2^ by setting *v*=0 in equation 4.

**Figure 4:**
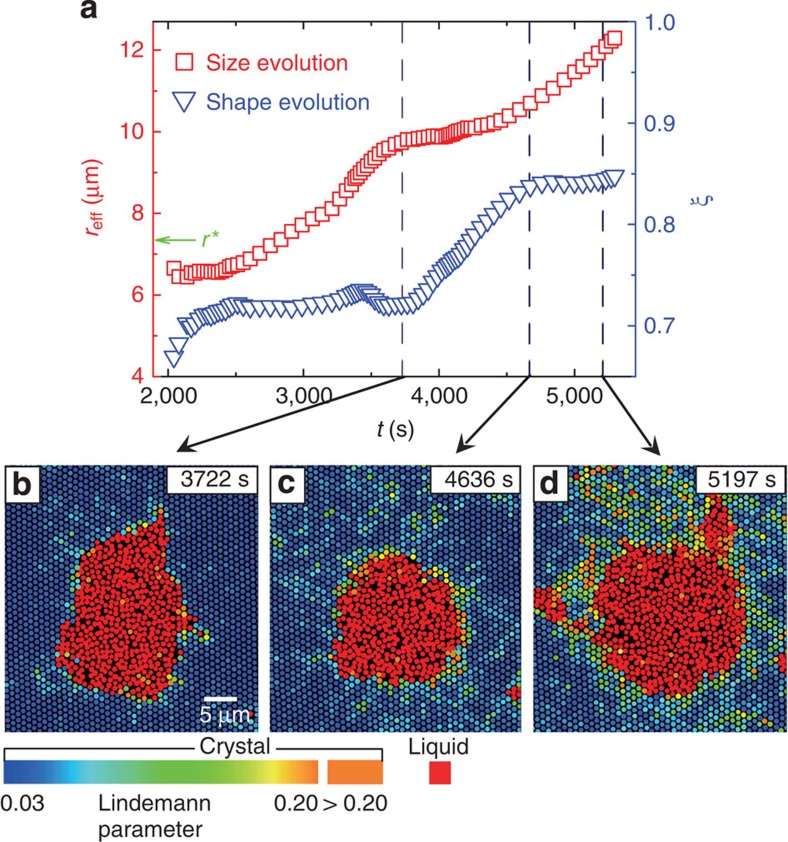
The coupling between nucleus shape and growth. (**a**) A typical evolution pattern of the nucleus size and 3D shape at weak superheating Δ*φ*=0.017 when *r*_eff_≲2r*. The nucleus grew little as it was transforming into a sphere but grew rapidly thereafter. (**b**–**d**) The real-space images correspond to the time labels in **a**. The heating light was turned on at *t*=0. The vibrational amplitude of each crystal-like particle is characterized by its Lindemann parameter, which is colour coded. Liquid-like particles in red are defined as those with low bond-orientational orders *ψ*<0.6 and divergent Lindemann parameter.

**Figure 5:**
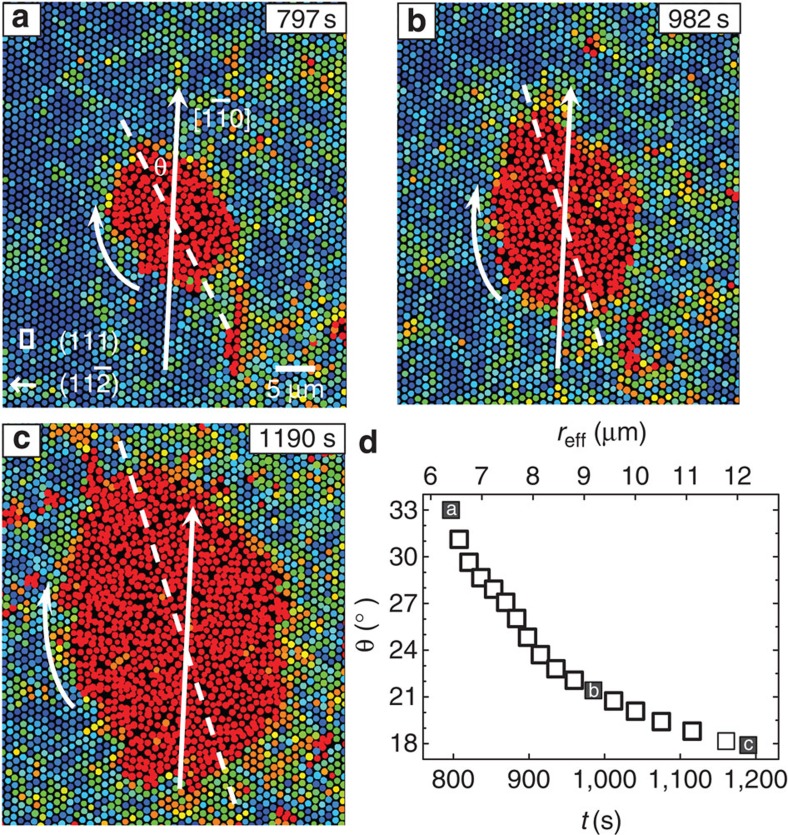
Nucleus rotation during its growth. (**a**–**c**) A typical nucleus growth process at intermediate superheating (Δ*φ*=0.041). The nucleus maintained its ellipsoidal shape during the growth, while its long axis rotated towards the 〈110〉 direction to minimize the surface energy. The liquid rotated through local melting and recrystallization without large particle displacements. Colours represent Lindemann parameters as in Fig. 4. (**d**) The time evolution of the nucleus orientation *θ* and the effective radius *r*_eff_. The black squares labelled with a, b, c correspond to panels **a**, **b**, **c**, respectively.

Next, we study the nucleus shape evolution and its effect on nucleus growth. Small liquid nuclei are more vulnerable to thermal fluctuations and less spherical, but not fractal-like as observed in colloidal crystallization^19^. The difference could be due to the fact that the dynamic heterogeneity of a supercooled liquid^48^ near vitrification could result in more irregularly shaped nuclei in crystallization, but a non-supercooled liquid in melting does not exhibit dynamic heterogeneity. For small non-spherical nuclei, we found that their growth rate is often not constant but is instead dependent on their shape (see Fig. 4 as an example). As the nucleus is becoming more spherical, which usually occurs at r*<*r*_eff_<2r* (Fig. 4 and Supplementary Movie 3), its growth slows down^49^. In addition, its centre of mass often exhibits discernible translational displacement along the 〈10〉 direction in the (111) plane (Supplementary Movie 4). When *r*>2r*, the nucleus usually grows at a constant rate while maintaining its shape (Fig. 4c,d and Supplementary Movie 3). It often becomes spherical before *r*>2r* at weak superheating as it has plenty of time to relax, but often maintains ellipsoidal shape at large size in the intermediate superheating. In Fig. 5, the ellipsoidal nucleus maintains its shape during the growth and rotates towards the 〈110〉 direction (Fig. 5 and Supplementary Movie 5) because the surface tension perpendicular to the 〈110〉 direction, that is, the (112) lattice plane, is minimal in hard-sphere fcc crystals^47^,^50^. Moreover, we found that the rotation speed decreases as it approaches the equilibrium orientation (Fig. 5d).

We found that the effects of surface tension and nucleus shape are less prominent at the early stage of nucleation at intermediate and strong superheating because their early stages are relatively short. The later stage of nucleation at intermediate and strong superheating exhibits fast growth (Fig. 3c), but we found that it is not mainly caused by the effects of surface tension and non-spherical shape. The surface tension effect is negligible when *r*_eff_>2r* and the growth rate reaches a constant as shown in Fig. 3a. The non-spherical shape leads to an enhancement in the growth rate of less than 10%, as all measured *ξ*>0.75 (that is, *v*(*ξ*)=*ξ*^−1/3^*v*≈1.1*v*) when Δ*φ*≲0.05. Further calculations in Supplementary Note 3 show that the shape fluctuation enhances the nucleus growth rate by less than 10%, and so it cannot explain the rapid growth above the weak superheating regime in Fig. 3c. Therefore, the high growth rate is most likely due to the multimer attachment as will be discussed in the section ‘Very Strong Superheating'.

### Strong superheating 0.05≲Δ*φ*≲0.06

**Figure 6:**
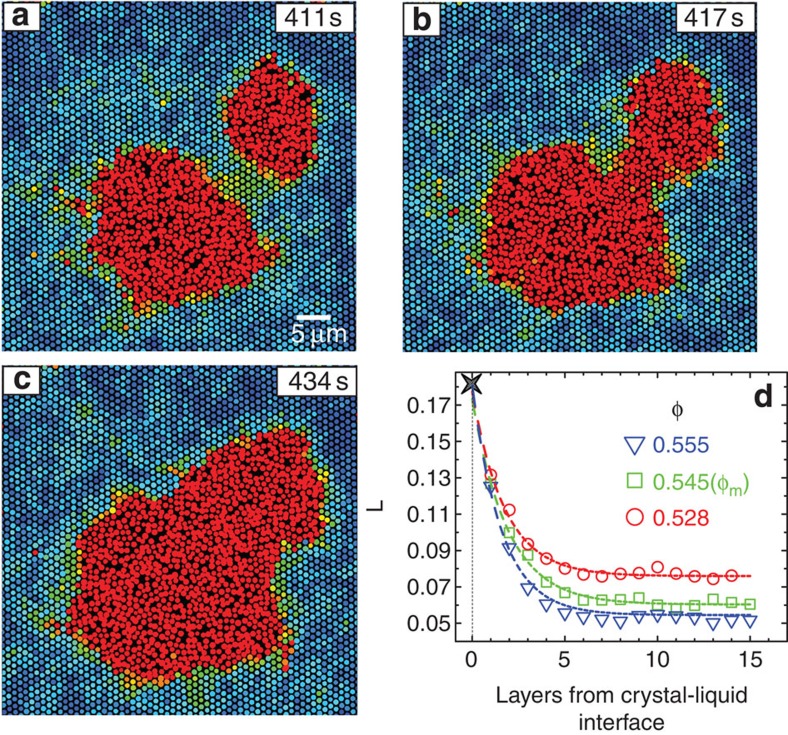
Nuclei coalescence at Δ*φ*=0.055. (**a**–**c**) When the two nuclei were less than eight layers of particles apart, a liquid channel developed and dramatically speeded up the coalescence. The Lindemann parameters are colour coded as in Fig. 4. (**d**) The averaged Lindemann parameter *L* as a function of distance from the crystal–liquid interface in crystals with *φ*>*φ*_m_, *φ*=*φ*_m_=0.545 and *φ*<*φ*_m_. The star represents the extrapolated value of *L* at the crystal–liquid interfaces. Dashed curves are the exponential fittings.

At strong superheating, the number density of nuclei is high and the frequent coalescence of nuclei is estimated to enhance the growth rate by about 100% from the experimental observations. The identity of the nucleus becomes ambiguous before and after the coalescence, and therefore the growth rate of individual nuclei at strong superheating is not measured in Fig. 3. The centres of the two post-critical nuclei exhibit no obvious displacements before and after coalescence. Before coalescence, we found that a strongly vibrational region was developed (Fig. 6a and Supplementary Movie 6) between the two post-critical nuclei when they were less than eight layers apart, which then melted into a liquid channel (Fig. 6b). The expansion of the liquid channel markedly (Fig. 6c) speeded up the nucleus growth during the coalescence. The eight-layer interaction range suggests that a liquid can affect the four neighbouring layers of lattices in the crystal. This is confirmed by the directly measured Lindemann parameter *L* in Fig. 6d. *L* exponentially decays to the bulk value within four layers of the crystal–liquid interface in both superheated (*φ*<*φ*_m_) and equilibrium (*φ*>*φ*_m_) crystals (Fig. 6d).

We obtained the Lindemann parameter *L* of the crystalline layer right next to the liquid by extrapolating the bulk values in Fig. 6d rather than direct measurement because the images at the solid–liquid interface were blurry. Interestingly, *L*≈0.18 for all the solid–liquid interfaces with different curvatures and at different volume fractions above or below the melting point (Fig. 6d). Note that *L*≈0.18 is an ensemble-averaged value at interfaces, and a superheated crystal could contain some bulk particles with *L*>0.18. Moreover, this universal value is close to our measured averaged bulk *L*=0.187 at the superheat limit Δ*φ*=0.09 (ref. 14). This result provides the first experimental support for the simulation result that surface *L* at the melting point is the same as bulk *L* at the superheat limit^51^. Our observations further show that the solid–liquid interfacial *L* is robust to different degrees of superheating and even weak supercooling. Therefore, the universal value of *L*=0.18 appears to be physically significant in both heterogeneous surface melting and homogeneous bulk melting.

### Very strong superheating 0.06≲Δ*φ*≲0.09

**Figure 7:**
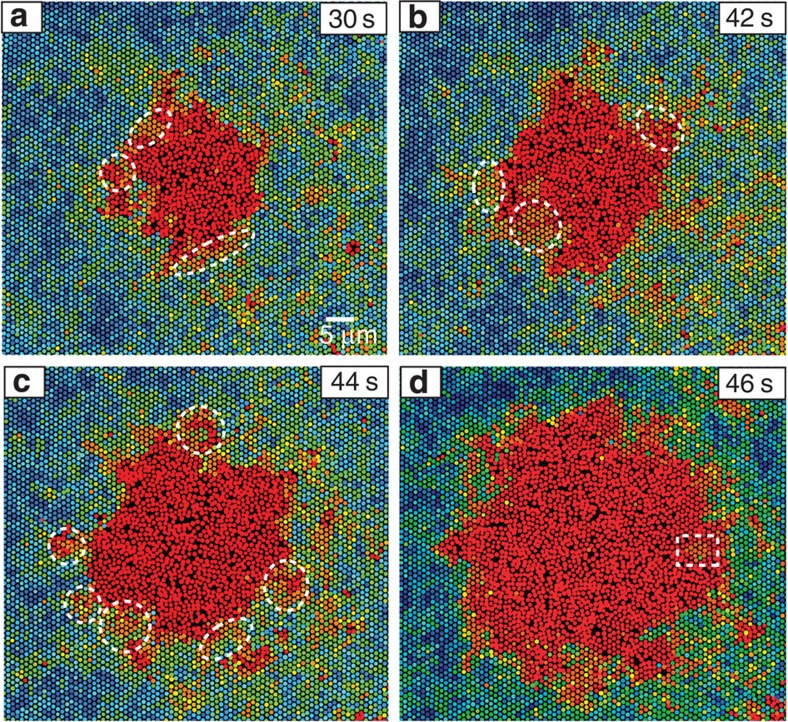
A typical nucleus growth process at very strong superheating Δ*φ*=0.07. Circles and ellipses mark the strong-vibration crystalline regions, which melt quickly. The rectangle in (d) shows a crystallite embedded in liquid. The Lindemann parameters are colour coded as in Fig. 4.

At very strong superheating, nuclei grow extremely quickly. *v*=2 μm s^−1^ is 30 times faster than those at intermediate superheating in Fig. 3c. Post-critical nuclei rapidly overwhelmed any small nuclei nearby, and therefore large nuclei coalescence was rarely observed. Coalescence of small subcritical nuclei was observed, but no liquid channel formed between them since they were too small. As superheating shifted from intermediate to strong, *D*′, *f*, 1−exp[(*μ*_s_−*μ*_l_)/*k*_B_*T*] and ξ in equations 3 and 4 all changed by less than 100%, and cannot account for the dramatic increase in *v*. We attribute the rapid growth to multimer attachment. In Fig. 7 and Supplementary Movie 7, each crystalline patch (in white dotted circle) transform to the liquid nucleus as a whole. Consequently, the nucleus growth takes a lot less time than the monomer attachment assumed in CNT. Strong vibrating regions were randomly generated near the crystal–liquid interface and turned into a liquid within a second. Consequently, the nucleus surface was lumpy (Fig. 7) and small crystallites (or liquid) could even be embedded in the liquid (or crystalline matrix), for example, the white dotted rectangle in Fig. 7d.

Multimer attachment may also occur in the intermediate and strong superheating regimes, but small, transient multimers involving several particles are difficult to observe in 3D. The multimer attachment at very strong superheating affects not only stage III of nucleation, but also the formation of the critical nucleus in stages I and II. We observed that new-born nuclei were already larger than the critical size due to the simultaneous melting of multiple particles (Supplementary Movie 8).

At the superheat limit Δ*φ*=0.09, the crystal grew unstable with vanishing induction time and started melting catastrophically from everywhere. Using the energy barrier 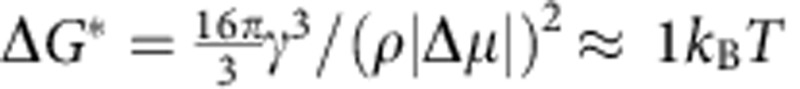 and Δ*μ*=0.35 *k*_B_*T* extrapolated from Supplementary Fig. 2 at the superheat limit, we obtain *γ*≈0.17 *kBT*/*σ*^2^, which is much smaller than 0.42 *k*_B_*T*/*σ*^2^ measured at weak superheating. This small *γ* and the rapid nucleus growth led to non-spherical nuclei with very lumpy surfaces as shown in Fig. 7.

## Discussion

By fabricating high-quality colloidal crystals with thermally sensitive particles and applying the local optical-heating technique, we were able to resolve the microscopic kinetics of post-critical nucleus growth in homogeneous melting. The size and shape of the liquid nuclei were monitored during the growth process at different degrees of superheating. When nuclei were small, the surface tension reduced the growth rate. We quantitatively took this effect into account in equation 4, which fits the experimental data in Fig. 3a,b nicely and can yield the solid–liquid surface tensions at different degrees of superheating. When the nucleus radius was more than twice the critical radius, the surface tension effect was negligible and the growth rate became a constant at each degree of superheating (Fig. 3a,b).

The whole superheating range 0<Δ*φ*<0.09 is divided into four regimes according to the mechanism enhancing the nucleus growth. (I) At weak superheating, that is, Δ*φ*≲0.025, the nucleus growth followed the revised WF law in CNT. The difference between melting and crystallization arises from the facts that particles can only attach to appropriate lattice sites of a crystalline nucleus in crystallization, but not necessary in melting, and the diffusion coefficient in the growth law is always measured from the liquid phase. Above the weak superheating regime, we observed the breakdown of some of the assumptions in CNT. (II) At intermediate superheating, that is, 0.025≲Δ*φ*≲0.05, the growth rate was higher than that predicted by the revised WF law. We found that the non-spherical nucleus shape and the shape fluctuation each contributed to enhancements of <10% even for small post-critical nuclei. (III) At strong superheating, that is, 0.05≲Δ*φ*≲0.06, nuclei coalescence further speeded up the nucleus growth, especially during the expansion of the liquid channel between two nuclei. (IV) At very strong superheating, that is, 0.06≲Δ*φ*≲0.09, we observed multimer attachment to nuclei for the first time, which markedly promoted the growth rate. Approaching the superheat limit, the nucleus surface became increasingly rough because of the low surface tension and the fast nucleus growth. The rich phenomena help to clarify how different effects affect the kinetics at different stages of nucleation under different degrees of superheating. They provide new guidance for further refinement of the nucleation theory especially for stronger superheating, as the current nucleation theory is based on many assumptions, most of which only hold at weak supersaturation.

In addition, we observed a universal value of the Lindemann parameter at the solid–liquid interfaces under all volume fractions and in the bulk at the superheat limit, indicating that it is a feature common to heterogeneous interfacial melting and homogenous bulk melting.

Our experimental method for melting can be similarly applied to crystallization, in which the growth kinetics of post-critical nuclei has not been studied at the single-particle level. The effects of a non-spherical shape and the shape fluctuation of nuclei might be similar in melting and crystallization, but the nuclei coalescence and the multimer attachment at deep saturation may not be obvious in crystallization due to the vitrification of liquid.

## Methods

### Sample preparation

By directly imaging the isolated particles that were stuck to the glass wall in the dilute suspension, we found that the diameter *σ* changed linearly in the experimental temperature range (Supplementary Fig. 4). We placed a temperature controller (Bioptechs) on either side of the sample, one on the × 100 oil-immersion objective lens and the other on the condenser, to avoid a temperature gradient in the *z* direction. The immersion oil was added between the condenser and the glass sample cell for good thermal contact. The temperature controllers were calibrated such that the grain boundaries of colloidal crystals on the top and bottom glass walls melted simultaneously, which indicates that the temperature difference was less than 0.1 °C in the *z* direction of the whole sample.

### Local optical heating

We superheated the interior of the crystal with a beam of light from the 100W, mercury lamp while retaining the ambient temperature below the melting point. The area under heating in the focal plane was usually set to ∼1.5 mm in diameter by adjusting the iris. In this way, the temperature in the central area 150 μm in diameter was uniform (<0.2 °C). We observed the sample in the transmissive mode of an upright microscope in order to avoid the direct exposure of the camera to the heating light. 0.2% by volume of non-fluorescent black dye (Chromatech-Chromatint black 2232 liquid) was added to the sample to absorb the heating light more efficiently. The dye appeared to have minor effects on the particle interaction and the phase behaviour. Paraffin films were placed in the light path so that the optical heating was uniform enough for the nucleation to start from a random place each time, that is, homogeneous melting. Experimentally, the heating effect can be easily calculated from 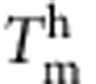, where 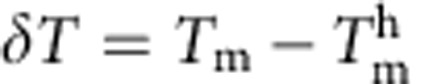 and *T*_m_ are the melting temperatures at a grain boundary with and without the optical heating, respectively. *δT* can be controlled by adjusting the light intensity and the dye concentration, and was usually set to 2 °C. The heating effect saturated 2 s after we turned on the heating light (Supplementary Fig. 5).

The induction time *t_i_*, defined as the time taken for the first post-critical nucleus to form^46^, becomes extremely long at very weak superheating. When Δ*φ*=*φ*_m_−*φ*≳0.015, we waited almost 2 h to obtain a post-critical liquid nucleus. When Δ*φ*<0.015, we burned a liquid nucleus with strong local heating to avoid the long induction time. We removed the paraffin films and minimized the aperture to heat a ∼(20 μm)^2^ region with *δT*≃4 °C so that a liquid nucleus could be produced rapidly. When the newly produced liquid nucleus was close to the critical size, we added paraffin films back and restored the aperture to its original size to heat a *π*(75 μm)^2^ region with *δT*=2 °C. The newly produced liquid nucleus was spherical as the aperture was circular (Supplementary Movie 1). It grew less spherical under thermal fluctuation after a few minutes.

## Author contributions

Y.H. and Z.W. conceived and designed the research. Z.W. carried out the experiment and data analysis with help from Y.P. Z.W. and F.W. did the theoretical analysis. Z.W. and Y.H. wrote the paper. Y.H. supervised the work. All authors discussed the results.

## Additional information

**How to cite this article:** Wang, Z. *et al*. Direct observation of liquid nucleus growth in homogeneous melting of colloidal crystals. *Nat. Commun*. 6:6942 doi: 10.1038/ncomms7942 (2015).

## Supplementary Material

Supplementary Figures, Supplementary Notes and Supplementary References Supplementary Figures 1-5, Supplementary Notes 1-3 and Supplementary References

Supplementary Movie 1 A demonstration of the local optical heating. The heating light was turned on at *t* = 2 s in the video. A filter was added in front of the CCD camera for better imaging at *t* = 3 s. The strong heating light burned a liquid nucleus at very weak superheating (Δ*ϕ* = 0.545 − *ϕ* = 0.013) and then was turned off at *t* = 9 s. (29× real time).

Supplementary Movie 2 A postcritical nucleus at Δ*ϕ* = 0.027 scanned in the z direction. (real time).

Supplementary Movie 3 A typical nucleus growth at weak superheating Δ*ϕ* = 0.017 (Fig. 3 of the main text). The non-spherical nucleus tends to become more spherical. During this process, the growth rate was slower. (19× real time).

Supplementary Movie 4 As the nucleus grew from *r*eff < 2r^*^ to *r*eff > 2r^*^, it became more spherical and the centre of mass exhibited a higher mobility than when the nucleus was small. (101× real time).

Supplementary Movie 5 A typical nucleus growth process of a liquid nucleus at intermediate superheating Δ*ϕ* = 0.041 (Fig. 4 of the main text). The nucleus maintained its shape during the growth and its long axis rotated towards the 〈110〉 direction. (27× real time).

Supplementary Movie 6 The coalescence of two postcritical nuclei at strong superheating Δ*ϕ* = 0.055 (Fig. 5 of the main text). A region of large Lindemann parameter and a liquid channel were developed between the two nuclei before coalescence. (4× real time).

Supplementary Movie 7 A typical nucleus growth process at very strong superheating Δ*ϕ* = 0.07 (Fig. 6 of the main text). Multiple particles in regions of large Lindemann parameter collectively transformed to liquid (multimer attachment) instead of individual particles diffusing into liquid one by one (monomer attachment) as CNT assumed. (real time).

Supplementary Movie 8 A typical nucleation process at very strong superheating Δ*ϕ* = 0.07. The nucleus was developed from a large strong-vibrating region. Multiple particles transformed into liquid so that the new-born nucleus was larger than the critical size. (real time).
