# Supplementary material for: Direct observation of liquid nucleus growth in homogeneous melting of colloidal crystals
Source: Nat Commun. 2015 Apr 21;6:6942. doi: 10.1038/ncomms7942 (PMC4411290; doi:10.1038/ncomms7942)
Supplement: Supplementary Figures, Supplementary Notes and Supplementary References — Supplementary Figures 1-5, Supplementary Notes 1-3 and Supplementary References [file ncomms7942-s1.pdf]

Supplementary Figures

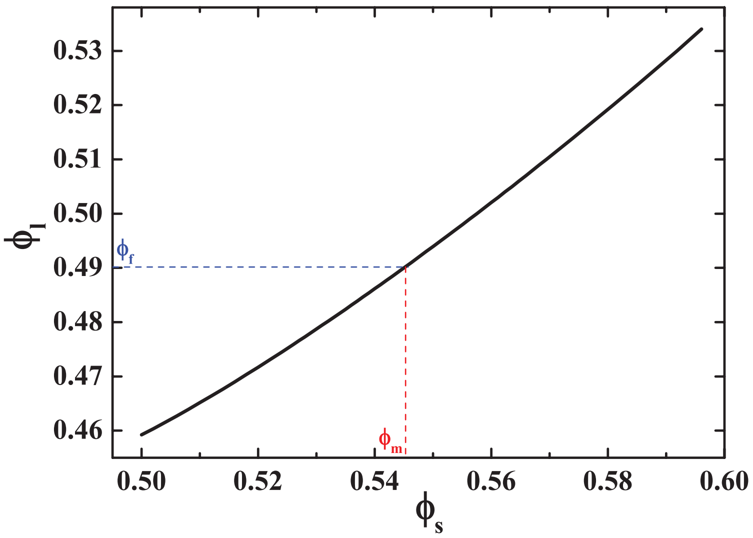

Supplementary Figure 1: ϕ_l vs. ϕ_s under the equipressure condition given by Supplementary Equations 2 and 3.

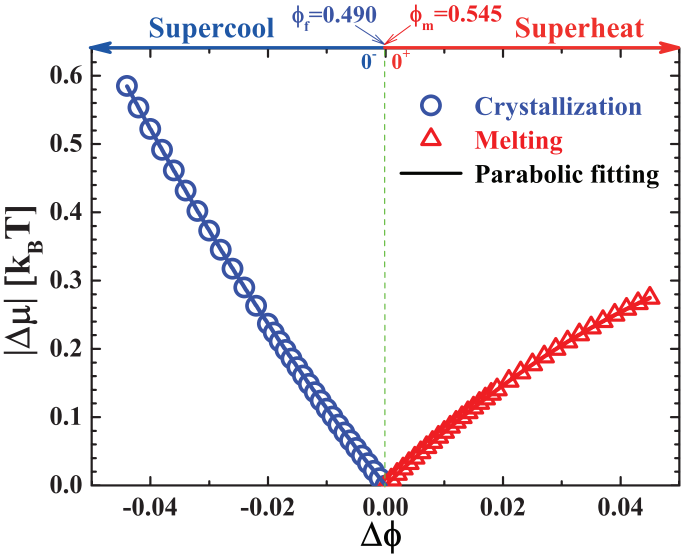

Supplementary Figure 2: $|\Delta\mu(\Delta\phi)| = |\mu_l - \mu_s|$ calculated from Supplementary Equations 1-3 is well fitted by $|\Delta\mu|/k_BT \approx -10.6\Delta\phi + 60.3\Delta\phi^2$ for crystallization and $|\Delta\mu|/k_BT \approx 8.30\Delta\phi - 48.9\Delta\phi^2$ for melting. The degree of supersaturation $\Delta\phi$ is defined as $\Delta\phi \equiv \phi - \phi_f$ for crystallization and $\Delta\phi \equiv \phi_m - \phi$ for melting.

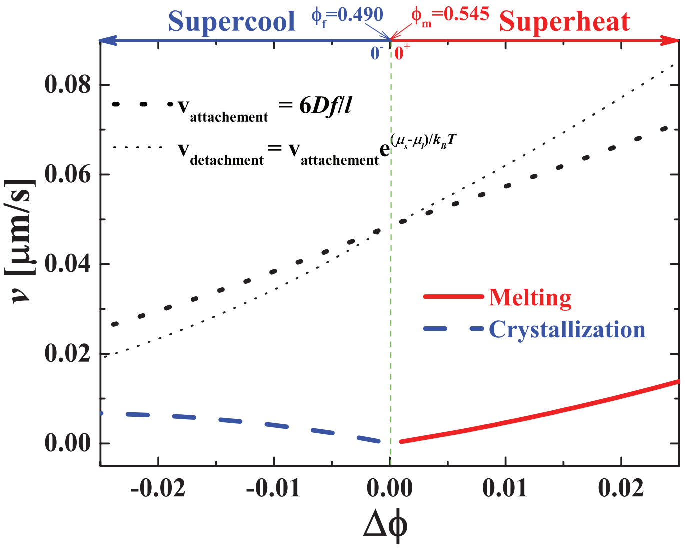

Supplementary Figure 3: Growth rate v as a function of the degree of superheating $\Delta(\phi)$ for both crystallization and melting based on Eq. 3 of the main text. $v(\Delta\phi)$ is a concave function for crystallization and a convex function for melting. The two terms in Eq. 3 of the main text are shown as the dotted curves.

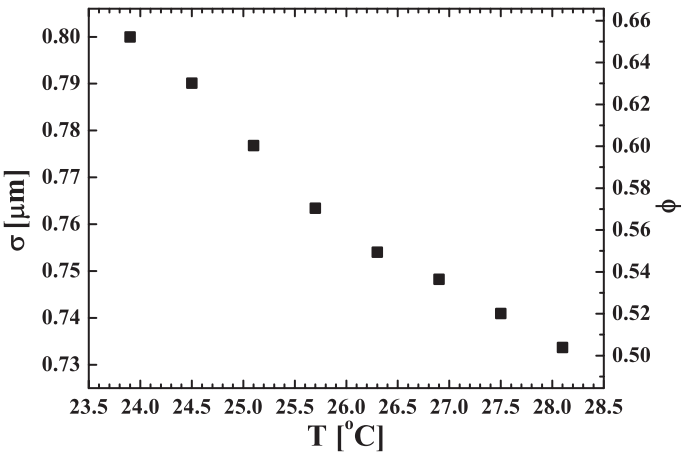

Supplementary Figure 4: The diameter σ of NIPA spheres and the corresponding volume fraction ϕ as a function of temperature.

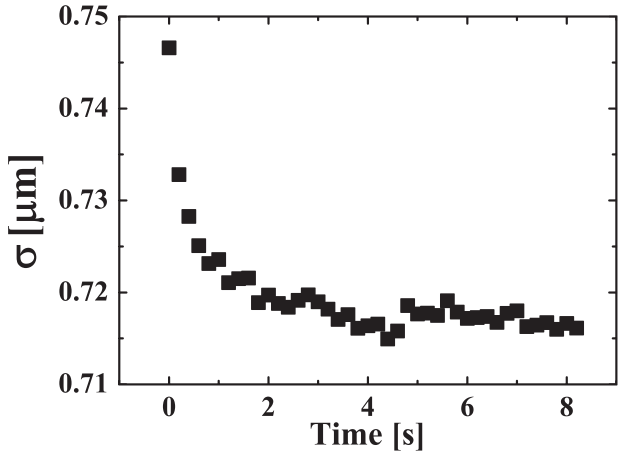

Supplementary Figure 5: The evolution of the diameter of NIPA particles stuck to the glass wall after the heating light was turned on at $t = 0$ s.

Supplementary Note 1: Calculation of the chemical potential difference $\Delta\mu$

In order to fit Fig. 3 of the main text, we need to substitute $\mu_s(\Delta\phi)$ and $\mu_l(\Delta\phi)$ into Eqs. 3 and 4 of the main text, where chemical potential μ_s applies to the superheated crystal and μ_l to the metastable liquid in the nucleus. When $0.490 < \phi < 0.545$, the solid with $\phi = 0.545$ and the liquid with $\phi = 0.490$ coexist at equilibrium, hence the chemical potential of the coexistence phase $\mu_{co}(\phi) = \mu_s(0.545) = \mu_l(0.490)$. However, if all hard spheres are liquid (or solid) at $0.490 < \phi < 0.545$, $\mu_l(\phi)$ and $\mu_s(\phi)$ for this metastable state are not available in literature. Directly extrapolating the values from the equilibrium regime to the metastable regime of $0.490 < \phi < 0.545$ will result in errors. Therefore we evaluate $\Delta\mu(\Delta\phi)$ using the equations of state (EOS)

of hard spheres which hold for broad ranges of ϕ including both equilibrium and metastable regimes.

$$|\Delta\mu(\Delta\phi)| = |\mu_s(\phi_s) - \mu_l(\phi_l)| = |(\mu_s(\phi_s) - \mu_{co} - (\mu_l(\phi_l) - \mu_{co}))| = |\mu_s(\phi_s) - \mu_s(0.545) - (\mu_l(\phi_l) - \mu_l(0.490))|$$
$$= \left|\int_{0.545}^{\phi_s} \frac{d\mu_s}{d\phi} d\phi - \int_{0.490}^{\phi_l} \frac{d\mu_l}{d\phi} d\phi\right| = \left|\int_{0.545}^{\phi_s} v_s \frac{dp_s}{d\phi} d\phi - \int_{0.490}^{\phi_l} v_l \frac{dp}{d\phi} d\phi\right| = \left|\int_{0.545}^{\phi_s} \frac{dp_s}{d\rho_s}\frac{d\phi}{\phi} - \int_{0.490}^{\phi_l} \frac{dp_l}{d\rho_l}\frac{d\phi}{\phi}\right|,$$
(Supplementary Equation 1)

where $\phi = \pi\rho\sigma^3/6$, the number density $\rho \equiv N/V \equiv 1/v$ and v is the volume per particle. In deriving Supplementary Equation 1, we used $d\rho/\rho = d\phi/\phi$ and the thermodynamic relation $v = (\partial\mu/\partial p)_T$. To evaluate $dp/d\rho$, we adopt Kolafa-Labík-Malijevský EOS for hard-sphere liquids [1]

$$p/(\rho k_B T) = 1 + 4x + 6x^2 + 2.3647684x^3 - 0.8698551x^4 + 1.1062803x^5$$
$$-1.095049x^6 + 0.637614x^7 - 0.2279397x^{10} + 0.1098948x^{14} - 0.00906797x^{22}$$
(Supplementary Equation 2)

and the revised Speedy EOS for hard-sphere fcc crystals [2]

$$p/(\rho k_B T) = \frac{3}{1-z} - \frac{a(z-b)}{z-c},$$ (Supplementary Equation 3)

where $x = \phi/(1-\phi)$, $z = \rho\sigma^3/\sqrt{2}$, $a = 0.620735$, $b = 0.708194$ and $c = 0.591663$. Supplementary Equations 2 and 3 fit the numerical simulation result $p/(\rho k_B T)$ with less than 4×10^{-5} error when $\phi_l < 0.539$ [2] and less than 10^{-5} error when $\phi_s > 0.497$ [1] respectively. Therefore they can be used to accurately evaluate $\Delta\mu(\Delta\phi)$ in our $0.490 < \phi < 0.545$. We substituted Supplementary Equations 2 and 3 into Supplementary Equation 1 and evaluated $\Delta\mu$ in Mathematica. ϕ_s in Supplementary Equation 1 can be accurately calculated from the measured number density and $\sigma(T)$, but ϕ_l cannot because the number density of liquid is not accurate due to the blurry image. Consequently we solved ϕ_l from the equipressure condition $p(\phi_l) = p(\phi_s)$ (Supplementary Figure 1) based on Supplementary Equations 2 and 3. ϕ_l is also used in the calculation of diffusion coefficient $D(\Delta\phi)$.

The numerical results $\Delta\mu(\Delta\phi)$ for both melting and crystallization are shown in Supplementary Figure 2 which can be well fitted by quadratic polynomials. The quadratic polynomial for melting was substituted into Eqs. 3 and 4 to fit Fig. 3c and 3b of the main text respectively. Note that it is necessary to use Supplementary Equations 2 and 3 fitted from the recent simulations because old EOSs for hard spheres are not accurate enough in the superheat regime [3]. We substituted $\mu_s(\Delta\phi)$ and $\mu_l(\Delta\phi)$ into Eq. 3 of the main text and obtained $v(\Delta\phi)$ in Supplementary Figure 3, which shows that the melting and crystallization branches are asymmetric around $\Delta\phi = 0$.

Supplementary Note 2: The effects of a non-spherical shape on nucleus growth

In Eq. 4 of the main text, we added the non-spherical parameter $\xi^{-1/3}$ in the prefactor and in the exponent to account for the effect of a non-spherical shape on nucleus growth. Here we show the detailed derivation. The free energy of a nucleus $\Delta G = S\gamma - V\rho_l|\Delta\mu|$, where $V = \frac{4}{3}\pi r_{\text{eff}}^3$ is the nucleus volume, $S = 4\pi r_{\text{eff}}^2 \xi^{-1/3}$ is the nucleus surface area and ρ_l is the number density of the liquid nucleus. At the critical size, ΔG attains its maximum. Hence by solving $\left.\frac{d\Delta G}{dr_{\text{eff}}}\right|_{r^*_{\text{eff}}} = 0$, we obtain the critical

size $r^*_{\text{eff}}(\xi) = \frac{2\gamma\xi^{-1/3}}{\rho\Delta\mu} = r^*\xi^{-1/3}$, where r^* is the critical radius for a spherical nucleus. Consequently r_{eff} in the exponent is replaced by $r\xi^{-1/3}$. $\xi^{-1/3}$ in the prefactor of Eq. 4 of the main text arises as follows. For the interface-reaction-limited growth, the volume increase is proportional to the surface ($\frac{dV}{dt} = kS$) for both spherical and non-spherical nuclei. For a non-spherical nucleus, $k4\pi r^2_{\text{eff}}\xi^{-1/3} = kS = \frac{dV}{dt} = k4\pi r^2_{\text{eff}}\frac{dr_{\text{eff}}}{dt}$, hence $v(\xi) \equiv \frac{dr_{\text{eff}}}{dt} = \frac{kS}{4\pi r^2_{\text{eff}}} = \xi^{-1/3}k = \xi^{-1/3}v_0$, where v_0 is the growth rate of a spherical nucleus.

Supplementary Note 3: The effects of shape fluctuation on nucleus growth

In the previous section, we showed that $v(\xi) = \left(\frac{S_{\text{sphere}}+\Delta S}{S_{\text{sphere}}}\right)v_0 = \xi^{-1/3}v_0$ for a non-spherical nucleus at a fixed ξ, where ΔS is the area additional to that of a perfect spherical nucleus with the same volume. However, neither ξ nor ΔS is fixed at a given degree of superheating, but follows a probability distribution under thermal fluctuations. The surface energy follows the Boltzmann distribution $e^{-\frac{\Delta S\gamma}{k_B T}}$, hence the probability of ΔS can be estimated as $P(\Delta S) = \frac{e^{-\frac{\gamma\Delta S}{k_B T}}}{\int_0^\infty e^{-\frac{\gamma\Delta S}{k_B T}}\,d\Delta S} = \frac{\gamma}{k_B T}e^{-\frac{\gamma\Delta S}{k_B T}}$ and the averaged growth rate is

$$\langle v(\xi)\rangle_\xi = \int_0^\infty v(\xi)P(\Delta S)d\Delta S = \int_0^\infty \left(\frac{S_{\text{sphere}} + \Delta S}{S_{\text{sphere}}}\right)v_0 \cdot \frac{\gamma}{k_B T}e^{-\frac{\gamma\Delta S}{k_B T}} \cdot d\Delta S = (1+\delta)v_0, \qquad \text{(Supplementary Equation 4)}$$

where $\delta = \frac{k_B T}{S_{\text{sphere}}\gamma}$. At intermediate superheating ($\Delta\phi \sim 0.04$), the measured $S^*_{\text{sphere}} = 26\,\sigma^2$ and the fitted $\gamma = 0.42\,k_B T/\sigma^2$ for a critical nucleus, thus $\delta = 9.1\%$. Note that this overestimates the effect of shape fluctuation since larger post-critical nuclei have smaller δ. Therefore the nucleus shape fluctuation contributes less than 10% to the fast growth rate at intermediate superheating in Fig. 3c of the main text.

Supplementary References

---

[1] Kolafa, J., Labík, S. & Malijevský, A. Accurate equation of state of the hard sphere fluid in stable and metastable regions. *Phys. Chem. Chem. Phys.* **6**, 2335–2340 (2004).

[2] Bannerman, M. N., Lue, L. & Woodcock, L. V. Thermodynamic pressures for hard spheres and closed-virial equation-of-state. *J. Chem. Phys.* **132**, 084507 (2010).

[3] Ackerson, B. J. & Schätzel, K. Classical growth of hard-sphere colloidal crystals. *Phys. Rev. E* **52**, 6448–6460 (1995).

[4] Kashchiev, D. *Nucleation: basic theory with applications* (Butterworth-Heinemann, 2000).
